# Persistent Carriers of Entamoeba Histolytica

**Published:** 1918-05

**Authors:** 


***Persistent Carriers of Entamoeba Histolytica.*** By S. Shepheard and D. G. Lillie. Abs. from the *Lancet*, April 6, 1918.

The authors treated 81 persistent carriers of Entamoeba histolytica with Chaparro Amargosa and Simaruba, administered by Nixon's method. All but one had had at least two courses of emetine bismuth iodide but still continued to pass either cysts or free E. histolytica in their stools; an interval of at least three weeks was allowed to elapse between the two treatments.

*Results.*

| Preparation | Cases treated | Cases "cured" |
|---|---|---|
| Chaparro, twigs and leaves . . . . . | 7 | 4 |
| " root-bark . . . . . . . . . | 30 | 11 |
| " root . . . . . . . . . . . | 33 | 16 |
| " bitter principle . . . . . . | 4 | 0 |
| Simaruba . . . . . . . . . . . . . . | 7 | 3 |
| | 81 | 34 |

When a first course failed, a second was found to be useless. All the preparations caused pain, vomiting, nausea, and increased motions, though the symptoms were not so severe as to interfere with treatment. No case with free E. histolytica in the stools was cured. The percentage of cures was higher among those who suffered no ill effects from the drug. Six cases were given drinks without enemas; of these three were cured.

